# Preterm-born children, screen time, and spectacle wear in the Longitudinal Preterm Outcome Project: a cohort study

**DOI:** 10.3389/fped.2025.1514413

**Published:** 2025-02-13

**Authors:** V. Iyer, M. L. A. de Kroon, C. C. W. Klaver, S. A. Reijneveld

**Affiliations:** ^1^ Department of Health Sciences, University Medical Center Groningen, Groningen, Netherlands; ^2^ Department of Child Health, TNO, Leiden, Netherlands; ^3^ Department of Public Health and Primary Care, Centre Environment & Health, KU Leuven, Leuven, Belgium; ^4^ Department of Ophthalmology, Erasmus Medical Center, Rotterdam, Netherlands; ^5^ Department of Epidemiology, Erasmus Medical Center, Rotterdam, Netherlands; ^6^ Department of Ophthalmology, Radboud University Medical Center, Nijmegen, Netherlands; ^7^ Institute of Molecular and Clinical Ophthalmology Basel, Basel, Switzerland

**Keywords:** gestational age, preterm birth, child, adolescent, spectacles, screen time

## Abstract

**Introduction:**

Preterm born children are at a higher risk for refractive errors. A long duration of screen time and activities with short working distance (≤30 cm) may further add to the increased risk. The aim of this study was to assess the separate and combined effects of preterm birth and screen time on spectacle wear among 5-year-olds and adolescents.

**Methods:**

We analyzed data from the community-based preterm cohort study, part of the Longitudinal Preterm Outcome Project (LOLLIPOP). Early preterm-born (EP < 32 weeks), moderately-late preterm-born (MLP 32–36 weeks) and full-term born (FT 38–42 weeks) children were followed. Spectacle wear and screen time were assessed by questionnaire at the age of 5 (*n* = 1,515) and at adolescence, ages 13–16, for a subsample (*n* = 227).

**Results:**

At age 5, the prevalences of spectacle wear were 7.8%, 7.6% and 3.2%, for EP, MLP, and FT children, respectively (*p* = 0.007); the risk of spectacle wear decreased by 7% for each additional week of gestational age. In adolescence, prevalences were 36.6%, 20.8% and 22.4%, for EP, MLP, and FT children, respectively (*p* = 0.12). We found no relationship between screen-time and spectacle wear or a combined effect with preterm birth at age 5 or adolescence.

**Conclusions:**

EP and MLP children have a significantly increased risk of spectacle wear at age 5, but not significantly at adolescence. At that age, the prevalences of spectacle wear were generally higher. We found no evidence for an association of screen time preterm birth with spectacle wear, and neither an impact of screentime on such an association.

## Highlights

• Early and Moderately-late preterm-born (EP and MP) children have a significantly increased risk of spectacle wear at age 5.
• At age 5, the risk of spectacle wear decreased by 7% for each additional week of gestational age.
• The prevalence of spectacle wear in adolescence was higher than at age 5; however, in adolescence the difference between preterm and term born children was not statistically significant.
• We did not find a significant association between screen time and spectacle wear in our cohort study.

## Introduction

Every year an estimated 15 million babies are born preterm, i.e., “*born before 37 completed weeks of gestation*” as defined by the World Health Organization (WHO) and this number is still rising (1). When compared to full-term born children (FT), both early preterm-born (EP, born before 32 weeks of gestation) and moderately-late preterm born (MLP, born between 32 and 36 weeks of gestation) are at a higher risk of impaired growth, cognitive, motor and visual development (2, 3). Studies have shown that the prevalence of refractive errors and visual morbidities is higher in preterm than in full-term adolescents (4). In preterm born children, the major ophthalmological abnormalities are refractive errors such as myopia, hypermetropia and astigmatism (5).

A long duration of screen time and activities with short working distance may further add to the increased risk of inducing refractive errors in preterm-born children (6). In the general pediatric population, several studies showed a relationship between (indoor) screen time and myopia, especially at a short distance (≤30 cm) (7–13). Early onset of myopia increases the risk of acquiring high myopia (i.e., <−6 diopters) and myopia-related complications later in life (10, 14, 15). Parental socioeconomic status (SES) and sex may play a role in the onset of myopia, with lifestyle factors such as reduced outdoor activities being an underlying mechanism (16, 17). However, findings on lifestyle trends are inconsistent (11–13, 15, 16, 18, 19). Studies show that there are sex-related differences in lifestyle and habits including screen time (20, 21).

As preterm-born children are at increased risk of acquiring refractive errors it is important to assess whether screen time plays an additional role in these children. Few studies have been published on the relationship between the exact gestational age (GA), assessed in weeks, and refractive errors in later life (5). Additionally, it has not been established if there is a relationship between screen time and refractive error among preterm-born, with a follow-up until adolescence. Refractive errors can be optically corrected with spectacle wear (or contact lenses). Our aim was therefore to assess the separate and combined effects of GA (in weeks) and screen time on spectacle wear at age 5 and at adolescence. We further examined the effects of SES and sex, as other relevant determinants of refractive errors (16, 17).

## Materials and methods

### Study population and setting

We analyzed data from the Longitudinal Preterm Outcome Project (LOLLIPOP) (3). The cohort comprised children born in the Netherlands between 2002 and 2003, and data have been collected on all preterm born at GA 36 weeks or less from 13 preventive child health care centers (PCHC), serving in total 45,446 children and further on all preterm born at 5 Neonatal Intensive Care Units (NICU, *N* = 548). This resulted in the LOLLIPOP cohort of 2,758 children in three groups: (a) Children <32 weeks (*n* = 352) (b) Children 32–36 weeks (*n* = 1,468) and as control (c) one full-term born child selected out of the PCHC database for every two preterm children included (*n* = 938 controls). These children were included when the children visited the PCHC at the age of 43–49 months after birth. Additionally, early preterm children were selected from the databases of the 5 NICUs, at the same ages (after removing the double sampled due to overlap of NICU and PCHC cohort, *N* = 548). Children were excluded if they had any congenital malformations, syndromes or congenital infections, resulting in a final cohort of 2,517 children. A detailed flow chart with the sampling procedures showing the inclusion of 2,517 infants has been published by Kerstjens et al. (22). For outcome analysis, a subsample of adolescents (at age 15; *N* = 831) from this cohort living in the three northern provinces of the Netherlands was invited to participate, between April 2017 and November 2018. The retention rates were 47.4% (173 invited), 33.0% (394 invited), and 31.1% (264 invited) for the three gestational age categories, respectively. Non-participation was due to decline for unknown reasons or not being traced. Out of the total of 294 responses 227 adolescents (46 EP, 111 MLP and 70 FT) provided data on spectacle wear.

### Procedure and measures

We obtained data via parent- and adolescent-reported questionnaires, with most questions derived from previous studies such as the Health Behavior in School-aged Children study, the TRAILS cohort study and the Netherlands Permanent Study on Living Settings (POLS) of Statistics Netherlands, the latter being the source of the question on adolescent use of spectacles; the questionnaire was piloted before use (23, 24). Spectacle wear, including wearing contact lenses at adolescence, was a dichotomous study outcome; data were derived from a parental questionnaire (yes vs. no) at age 5 and at adolescence.

Gestational age (GA) at birth was an independent variable in our study. GA categories were defined as EP (less than 32 weeks), MLP (32–36 weeks) and FT (38–42 weeks). GA had routinely been assessed with early ultrasound measurements between 10^0^ (10 weeks and 0 days) and 12^6^ (12 weeks and 6 days) of gestation according to protocol (25). GA was registered in the medical charts at the onset of the LOLLIPOP cohort. In the regression analyses we used GA as a categorical and as a continuous variable (in weeks). Additionally, we centered GA as continuous variable for analyzing the relationship with screen time.

Screen time at a short distance was obtained from the parental questionnaire and the questions were categorized according to WHO guidelines ranging between no screentime to more than 2 h a day. Screen time at a short distance (mobile phone or laptop), defined as the use of screens at ≤30 cm was categorized into low duration (<30 min per day) vs. high duration (≥30 min per day). In adolescence short-distance screen time was scored as the number of days spent on screens (mobile or video games); responses were subsequently categorized into low exposure (0–4 days a week) and high exposure (≥4 days a week).

Covariates were parental socio-economic status (SES) and sex. SES was a composite variable based on the education and occupational level of both parents and the family income, derived from a parental questionnaire and categorized in low, medium and high (25). Measures were standardized to a *z*-score. Scores below the 25th percentile were considered as a low socioeconomic status and above the 75th percentile as a high socioeconomic status (25). Finally, birth weight and sex were extracted from the medical charts of the preventive child health care organizations.

### Statistical analysis

First, we examined the background characteristics across the GA groups. We tested differences using Chi Square tests and one-way ANOVA. Second, we assessed the relationships of GA and screen time with spectacle wear at age 5, using univariable logistic regression analyses. Next, we adjusted these analyses for parental SES and sex using multivariable logistic regression analyses. Finally, we assessed the combined effect of screen time and GA, adjusting for the same covariates (i.e., SES and sex). We repeated these analyses for adolescence. All analyses were performed using IBM SPSS version 28.

## Results

### Background characteristics

**Figure 1:**
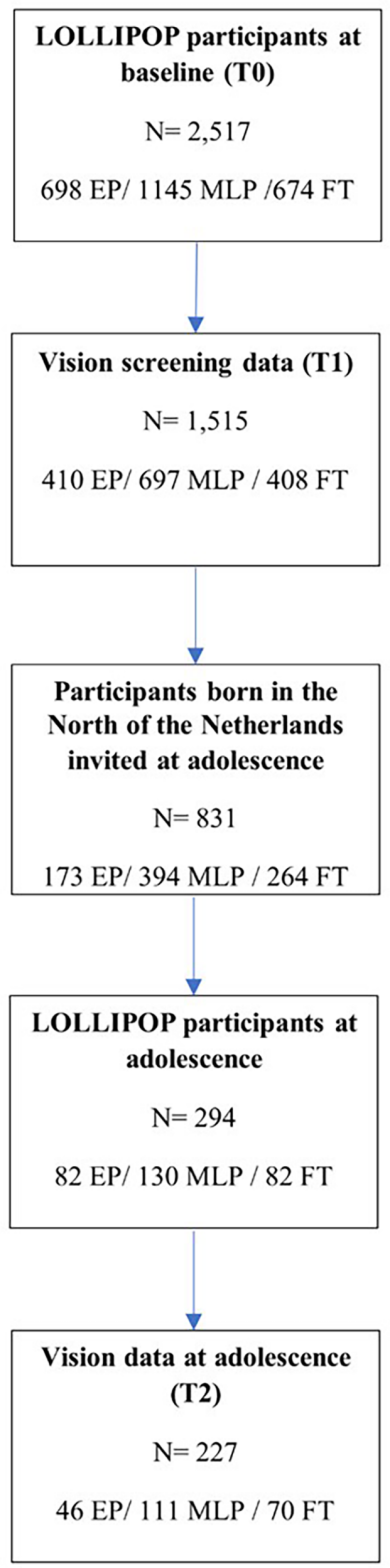
Flow of participants.

Of the 2,517 children from the LOLLIPOP study, we included 1,515 5-year-olds for our study, as only those children were screened for vision (Figure 1, Table 1A). Of these, 410 were EP, 697 MLP and 408 FT born (Figure 1, Table 1A). Regarding adolescents, 831 were invited to participate, and vision data was only available on 227: 46 EP, 111 MLP and 70 FT born (Figure 1, Table 1B).

**Table 1A:** Participant and behavioral characteristics of early preterm, moderately-late preterm and full-term born 5-year-olds (*N* = 1,515) in the LOLLIPOP cohort.

Characteristics	Early preterm (<32 weeks)	Moderately-late preterm (32–36 weeks)	Full-term (38–42 weeks)	*p*-value
	*N* = 1,515	*N* = 410	*N* = 697	*N* = 408
Gestational age, in weeks (mean; SD)	29.3 (1.66)	34.0 (1.04)	39.6 (1.00)	<0.001*
Weight at birth, in grams (mean; SD)	1,286.6 (384.8)	2,251.05 (458.9)	3,565.0 (479.5)	<0.001*
Male (*N*, %)	204 (49.8%)	402 (57.7%)	190 (46.6%)	<0.001**
Socioeconomic status^a^ (SES in percentile, p; *N*, %)				
Low (<25 p; 316, 22.1%)	80 (21.1%)	165 (24.8%)	71 (22.5%)	0.10**
Middle (25–75 p; 735, 51.3%)	189 (49.9%)	331 (49.8%)	216 (55.4%)	
High (>75 p; 382, 26.7%)	110 (29.0%)	169 (25.4%)	103 (26.4%)	
Spectacle wear	32 (7.8%)	53 (7.6%)	13 (3.2%)	0.007**
Screentime at a close distance^b^ (≤30 cm) ≥30 min day	133 (32.7%)	233 (33.8%)	166 (40.8%)	0.027**

^a^ Missing: 81.

^b^ Missing: 12.

* One-way ANOVA.

** Chi-square test.

**Table 1B:** Participant and behavioral characteristics of early preterm, moderately-late preterm and full-term born adolescents (*N* = 227) in the LOLLIPOP cohort.

Characteristics	Early preterm (<32 weeks)	Moderately-late preterm (32–36 weeks)	Full-term (38–42 weeks)	*p*-value
	*N* = 46	*N* = 111	*N* = 70	*N* = 227
Gestational age, in weeks mean (SD)	29.2 (2.13)	33.9 (1.09)	39.7 (0.89)	<0.001*
Weight at birth, in grams (mean; SD)	1,284.0 (458.65)	2,178.8 (516.9)	3,543.9 (444.5)	<0.001*
Age at follow-up mean (SD)	14.93 (0.75)	15.72 (0.54)	15.46 (0.55)	<0.001*
Sex, males (*N*, %)	19 (41.3%)	59 (53.2%)	29 (41.4%)	0.21**
Socioeconomic status^a^ (SES in percentile, p; *N*, %)				
Low (<25 p; 39, 19%)	8 (22.9%)	23 (21.9%)	8 (12.3%)	0.57**
Middle (25–75 p; 113, 55.1%)	19 (54.3%)	56 (53.3%)	38 (58.5%)	
High (>75 p; 53, 25.9%)	8 (22.9%)	26 (24.8%)	19 (29.2%)	
Spectacle wear^b^	15 (36.6%)	22 (20.8%)	15 (22.4%)	0.121**
Social media^c^ ≥4–5 days week	35 (85.4%)	96 (90.6%)	62 (92.5%)	0.47**
Online games^d^ ≥4–5 days week	11 (27.5%)	26 (25.2%)	15 (22.4%)	0.83**
Social media & gaming^e^ ≥4–5 days week	39 (95.1%)	102 (95.3%)	64 (95.5%)	1.00**

^a^ Missing: 22.

^b^ Missing: 13.

^c^ Missing: 13.

^d^ Missing: 17.

^e^ Missing: 12.

* One-way ANOVA.

** Chi-square test.

### Spectacle wear and screen time

**Figure 2:**
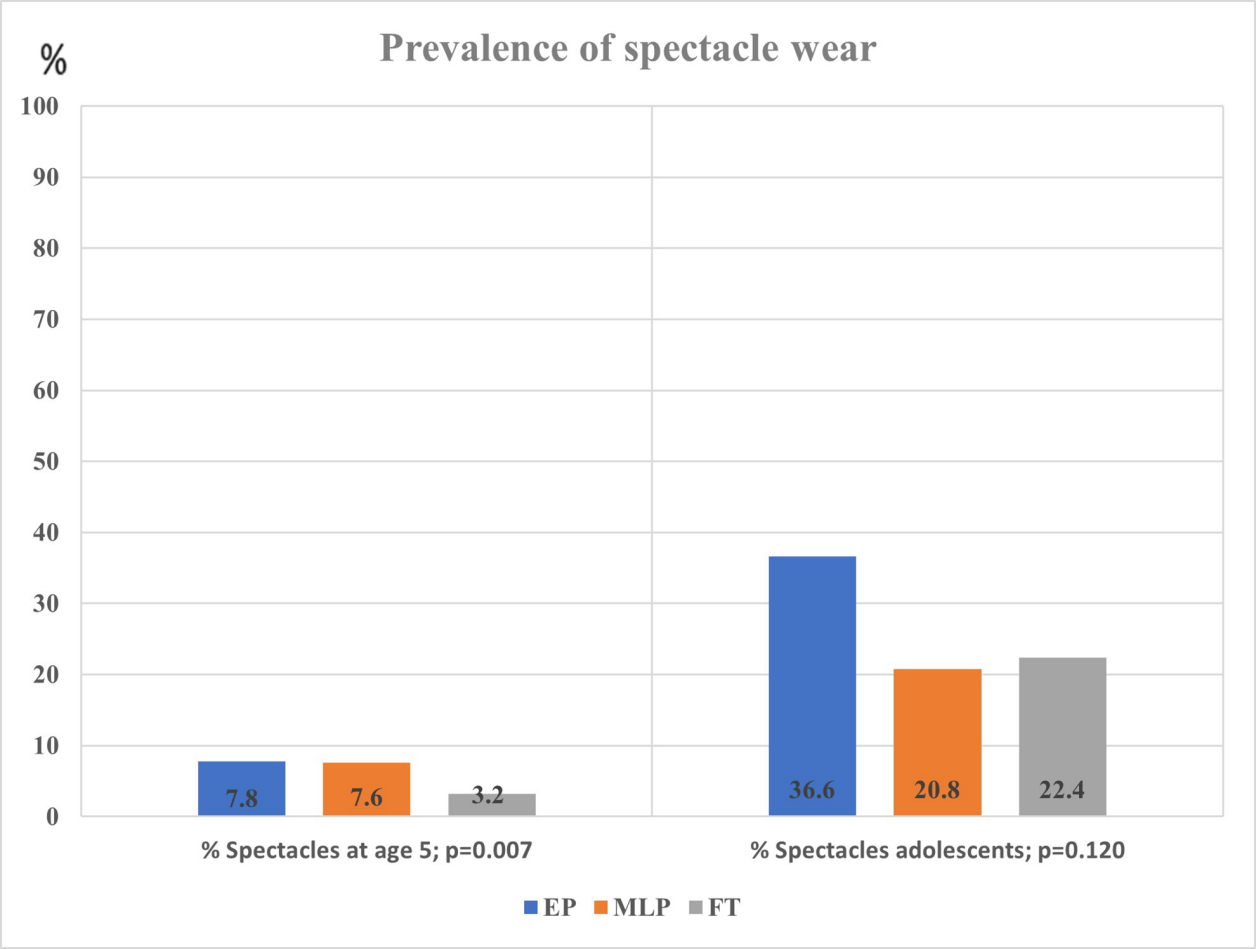
Spectacle wear at age 5 and at adolescence by gestational age group.

At age 5 the prevalence of spectacle wear was significantly different between the three GA categories, EP 7.8%, MLP 7.6% and FT 3.2% (*p* = 0.007; Table 1A, Figure 2). In the regression model sex was not significantly related to spectacle wear (*p* = 0.11). Table 2 shows that, after adjustment for sex, SES and screen time, GA as a continuous variable was related to spectacle wear at age 5 (odds ratio, OR 0.93, 95% confidence interval, CI 0.88; 0.98). We found a significant difference in (short distance) screen time between the groups EP, MLP and FT (*p* = 0.027; Table 1A). Children in the FT group showed the highest screen time. Screen time was not significantly associated with spectacle wear at age 5, neither in the univariable nor in the multivariable adjusted regression analyses. Additionally, no combined effect of gestational age (GA) and screen time was found (Table 2).

**Table 2:** Relationship between centred gestational age GA (in weeks) and/or screentime with spectacle wear (dichotomized) at age 5 (*N* = 1,515) and in adolescence (*N* = 214), odds ratios (95% confidence intervals, CI).

	Model 1a, crude model Independent variable	Model 1b: Model 1a, adjusted for sex and SES	Model 2a Combined model	Model 2b Model 2a, adjusted for sex and SES
At age 5				
GA in weeks (CI)	0.93 (0.88; 0.98)**	0.93 (0.88; 0.98)**	0.93 (0.89; 0.98)*	0.93 (0.88; 0.99)*
Screen time at a short distance	1.03 (0.67; 1.59)	1.04 (0.66; 1.62)	1.06 (0.69; 1.63)	1.06 (0.68; 1.66)
At adolescence				
GA in weeks (CI)	0.96 (0.89; 1.04)	0.97 (0.89; 1.05)	0.96 (0.89; 1.04)	0.97 (0.89; 1.06)
Screen time at a short distance	1.31 (0.27; 6.36)	1.18 (0.24; 5.89)	1.31 (0.27; 6.40)	1.18 (0.24; 5.88)

Crude model (model 1a) includes one independent variable, i.e., GA (in weeks), or screen time at short distance. Model 1a is model 1, adjusted for covariates. Combined model 2a includes GA and screen time at short distance. Combined model 2b, is the combined model adjusted for the covariates. Covariates are parental SES (socioeconomic status) and sex.

* *p* < 0.05.

** *p* < 0.01.

*** *p* < 0.001.

In adolescence, the prevalence of spectacle wear and/or contact lenses was higher than at age 5, and differed between the EP, MLP and FT born children (36.6%, 20.8% and 22.4%, respectively), but without statistical difference. The EP had >10% higher prevalence of spectacle wear than MLP and FT. However, as numbers were low, this difference did not reach statistical significance (*p* = 0.12; Table 1B, Figure 2). Regarding spectacle wear, significant combined relationships were not found between GA and screen time (Table 2).

## Discussion

We found that a shorter GA was related to a higher prevalence of spectacle wear at age 5, showing a decrease in the risk of spectacle wear by 7% for each additional week of gestational age. This relationship was not found in adolescence. Moreover, at both ages neither screen time nor the combined effects of GA and screen time showed a significant relationship with spectacle wear.

The higher prevalence of spectacle wear in 5 year old EP and MLP born children (7.8% and 7.7%, respectively) and the risk of spectacle wear decreasing by 7% for each extra week of gestational age are in line with findings in an earlier study (26). That study which investigated visual impairment after preterm birth showed that preterm birth was associated with a 2.4-fold increase in prevalence of refractive errors compared with FT born children. However, in this study gestational age was not studied as a continuous variable, but has been categorized as very preterm, moderately preterm, late preterm and term birth (in weeks) (26). A different study, performed in the United Kingdom, also showed a higher prevalence of spectacle wear in EP born children compared to term born children at the age of 7: 12.8% and 4.3%, respectively (27). However, that study neither specified preterm birth in weeks, nor included MLP born children. In summary, our results support earlier findings that prematurity increases the risk of spectacle wear and adds knowledge regarding the additional risk on spectacle wear for gestational age per week.

Among adolescents, spectacle wear varied between the EP, MLP and FT born children (36.6%, 20.8% and 22.4%, respectively), but these differences were not statistically significant. This lack of statistical significance may first be due to the limited sample size at adolescence (410 EP 5-year-olds vs. 46 EP adolescents) resulting in a lower statistical power. A second explanation for the higher frequency of glasses at adolescence compared to age 5 may be the natural occurrence of emmetropization, i.e., the shift towards emmetropia at school age, and a relatively equal risk of myopia development thereafter (28, 29). It has been shown that particularly moderate and high hypermetropia (refractive error ≥+2D) are more common in prematurely born children compared to full-term born children (30). A recent study reveals valuable insights concerning the development of refractive errors, including myopia, between the ages of 6–15 years (31). As individuals age, there is an observed increase in the proportion of myopes (31, 32).

Prevalences of spectacle wear at adolescence in our study varied between 22.4% and 36.6% and were much higher than at age 5. These prevalences seem to be comparable to those found in other studies (33). A German survey (2003–2006) showed that the prevalence of spectacle wear among adolescents (between the age of 14–17 years) from the general population was around 29.2% (30). However, in that study prevalences were not assessed related to GA. Additionally, it should be noted that prevalences might be underestimated because of visual undercorrection (inadequate visual correction or compliance), as was found in two studies in the general population and among adolescents who were both premature and underweight at birth (34, 35). This may imply that the real prevalences of spectacle wear in adolescents found in our study are even higher.

Our study did not find a relationship between screen time and spectacle wear, neither at age 5 nor at adolescence. These findings contrast with other studies. For instance, Irish school children who spent >3 h a day on a screen were more often myopic (36). Additionally, in a Dutch study among 9-year-olds in Rotterdam a mean computer use of 5.2 h/week was significantly associated with myopia (9, 37). Dutch teenagers used their smartphone almost 4 h a day and >20 min of continuous use was associated with more myopia (9). An explanation for the differences between our findings and those of other studies may be the low cut-off values we used (none to less than 30 min screentime) and our use of dichotomized answer categories for the registration of screen time in the LOLLIPOP database. Other studies had a higher number of categories which allowed registration of screen times up to 3 h/day (9, 36). Our registration in only two strata constrained the study of a dose-response relationship for screen time (38). In summary, our study did not replicate a significant relation between screen time and spectacle wear (12, 36, 39).

Our study had both strengths and limitations. A strength of our study is that this is the first study to show the association between GA as a continuous variable and spectacle wear as an outcome at short-term among 5-year-olds and at long-term in adolescents in a unique community-based birth cohort. Some limitations should also be mentioned. First, the relatively low response of the adolescents in the follow-up assessments could have created selection bias (40). The participatory sample included slightly more females, children born EP and children from a high SES. This might have introduced some bias although we do not expect that the relationship found between preterm birth and spectacle wear in adolescence is related to participation in the follow-up assessments. Second, information on screen time may have been somewhat imprecise as we used parent and adolescent report and had only dichotomized answer categories available for study. This may have led to underestimation of the real associations. Future studies should not only include more precise assessments but also cumulative effects of screen time on spectacle wear, enabling dose response studies.

## Conclusion

Preterm-born children had a significantly increased risk of spectacle wear at age 5, but not significantly at adolescence. At that age, the prevalences of spectacle wear were generally higher. Increased screen time at short distance could not be established as a risk factor for spectacle wear in our study, neither at age 5 nor at adolescence. More research in a larger population is needed for investigating the influence of lifestyle in both preterm and term born children, focusing on the effects of indoor activities (including screen time) on refractive errors in the short and long term.

## Data Availability

The datasets presented in this article are not readily available because the data that support the findings of this study are available from the LOLLIPOP study data access committee, consisting of the principal investigators of the project. Restrictions apply to the availability of these data as the participant consent for the collection of the data did not explicitly or implicitly include details of sharing their anonymized data. Data are however available from the authors upon reasonable request and with permission of the access committee reached via t.hoekstra@umcg.nl, manager of the data repository of the Department of Health Sciences and secretary of the access committee. Requests to access the datasets should be directed to T. Hoekstra, t.hoekstra@umcg.nl.
